# A Laminated Microfluidic Device for Comprehensive Preclinical Testing in the Drug ADME Process

**DOI:** 10.1038/srep25022

**Published:** 2016-04-28

**Authors:** Fan An, Yueyang Qu, Yong Luo, Ning Fang, Yang Liu, Zhigang Gao, Weijie Zhao, Bingcheng Lin

**Affiliations:** 1 State Key Laboratory of Fine Chemicals, Department of Chemical Engineering, Dalian University of Technology, 2 Linggong Rd., Dalian 116024, China; 2 School of Pharmaceutical Science and Technology, Dalian University of Technology, 2 Linggong Rd., Dalian 116024, China; 3 Department of Chemistry, Georgia State University, Atlanta, GA, 30302, USA; 4 Dalian Institute of Chemical Physics, Chinese Academy of Sciences, Dalian, China

## Abstract

New techniques are urgently needed to replace conventional long and costly pre-clinical testing in the new drug administration process. In this study, a laminated microfluidic device was fabricated to mimic the drug ADME response test *in vivo*. This proposed device was loaded and cultured with functional cells for drug response investigation and organ tissues that are involved in ADME testing. The drug was introduced from the top of the device and first absorbed by the Caco-2 cell layer, and then metabolized by the primary hepatocyte layer. It subsequently interacted with the MCF-7 cell layer, distributed in the lung, heart and fat tissues, and was finally eliminated through the dialysis membrane. Throughout this on-chip ADME process, the proposed device can be used as a reliable tool to simultaneously evaluate the drug anti-tumor activity, hepatotoxicity and pharmacokinetics. Furthermore, this device was proven to be able to reflect the hepatic metabolism of a drug, drug distribution in the target tissues, and the administration method of a drug. Furthermore, this microdevice is expected to reduce the number of drug candidates and accelerate the pre-clinical testing process subject to animal testing upon adaptation in new drug discovery.

The traditional new drug discovery process is extremely costly, time-consuming and inefficient, and consequently, it has retarded fast advances in the drug industry and improvements in human health. For example, developing a new drug usually requires an average of 10–15 years^1^ and costs from $1.5 billion to more than $1.8 billion^2^. Unfortunately, FDA-approved drugs are not guaranteed to meet market needs. The high cost and long development periods are primarily due to the extremely high failure rate of drug candidates in animal tests and clinical trials. According to the 2015 profile of Pharmaceutical Research and Manufacturers of America (http://www.phrma.org/sites/default/files/pdf/2015_phrma_profile.pdf), only 6 of a few hundred drug candidates entered the trial stage, and only one was approved by the FDA. Significantly narrowing down the drug candidates subject to animal tests and clinical trials would be an effective way to revitalize the drug discovery industry.

Drug candidates that survive the preclinical testing may ultimately fail during the animal testing or clinical trial stage because the data obtained from *in vitro* drug screening platforms, such as a 96-well plate, cannot be extrapolated to the real body of animals or humans. In the 96-well plate test, drug candidates interact directly with the target cells. However, a drug candidate experiences a complex *in vivo* ADME process (absorption, metabolism, distribution and excretion). Differences in the physiological environment, concentration and even molecular formula of the drug candidates *in vivo* may lead to significant variations in their ADME process, which strongly affect the final efficacy and toxicity of the drug candidate. Traditional *in vitro* drug screening tools cannot effectively recreate the ADME process of a drug candidate in the body and consequently frequently yield biased screening results.

In this study, we attempted to recreate the ADME process *in vitro* and develop a platform for drug screening. To this end, we selected a microfluidic chip as the new ADME screening platform because it may allow the co-culture of multiple types of functional cells and a comprehensive analysis of cells, drug candidates and their metabolites. To date, a variety of functional cells^3^,^4^,^5^,^6^,^7^,^8^,^9^,^10^ have been successfully cultured inside the micro-channels in a microfluidic chip. The co-culture of multiple types of cells in a number of microfluidic chips has also been reported^11^,^12^,^13^,^14^. Moreover, cell chips have been intensively used for drug screening, either for toxicity^15^, efficacy^16^ or pharmacokinetics^12^. They have even been transformed into “organ-on-a-chip^17^”, “body-on-a-chip^11^” and “human-on-a-chip^18^” devices that have demonstrated a strong potential to revolutionize drug discovery. We herein attempted to develop a new type of cell co-culture chip for preclinical testing featuring an artificial ADME process.

## Results

### Device fabrication

**Figure 1:**
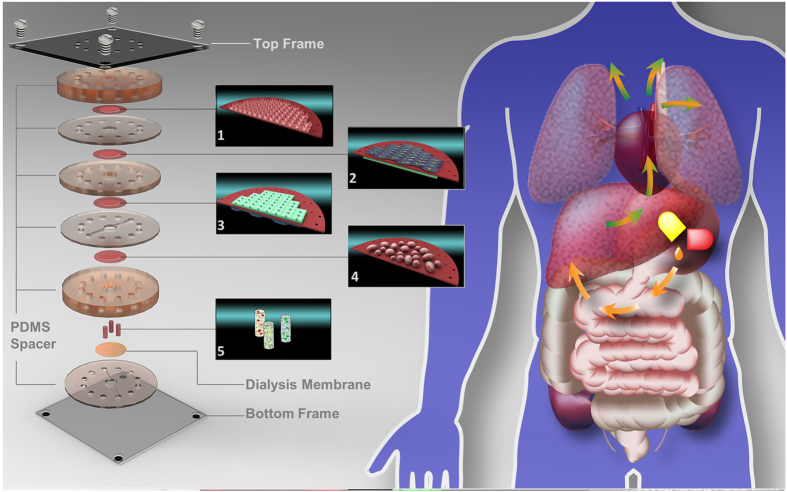
The exploded view of the laminated microfluidic device (left part) mimicking the ADME process of an oral drug *in vivo* (right part was illustrated by the first author, Fan An). (1) illustration of the Caco-2 cell layer on the PC membrane; (2) illustration of HUVEC (gray) and hepatocyte (green) layers on the membranes; HUVECs are on the top, and hepatocytes are on the bottom. (3) illustration of HUVEC (gray) and hepatocyte (green) layers on the membranes, hepatocytes are on the top, and HUVEC are on the bottom; (4) illustration of the MCF-7 cell layer on the PC membrane; (5) illustration of the lung, heart and adipose tissues located in the 5^th^ PDMS spacer from the top.

As demonstrated in Fig. 1, the main structure of this multilayered microfluidic device consisted of six PDMS (Dow Corning, USA) plates with a diameter of 30 mm, from top to bottom and thicknesses of 6 mm, 1.2 mm, 2.4 mm, 1.2 mm, 4.8 mm, or 1.2 mm. Microchannels (0.2 mm deep, 0.8 mm wide, and 18 mm long) were fabricated, from the top to bottom, in the 1^st^, the 4^th^ and the 6^th^ PDMS plates for cell culture medium perfusion. The PDMS plates for these structures, which were developed by Whitesides G.M. *et al*.^19^, were fabricated by soft lithography using SU8 3035 (Microchem, USA) as the template. Peripheral holes (1-mm diameter) were punctured in the PDMS plates to arrange tubing. Additionally, 5 mm diameter holes were made in the center of the each PDMS plate to facilitate connections between the cells and tissues between layers. Porous polycarbonate membranes with a 1-μm pore size were placed between PDMS plates for on-chip cell culture. After a careful alignment along the vertical direction, the PDMS plates were superimposed with the top and bottom PMMA plates and fastened with screws. The entire microfluidic device measured 4 cm × 4 cm × 2.1 cm (Figure S1 in S.I.).

### Characterization of cell layers and tissues

**Figure 2:**
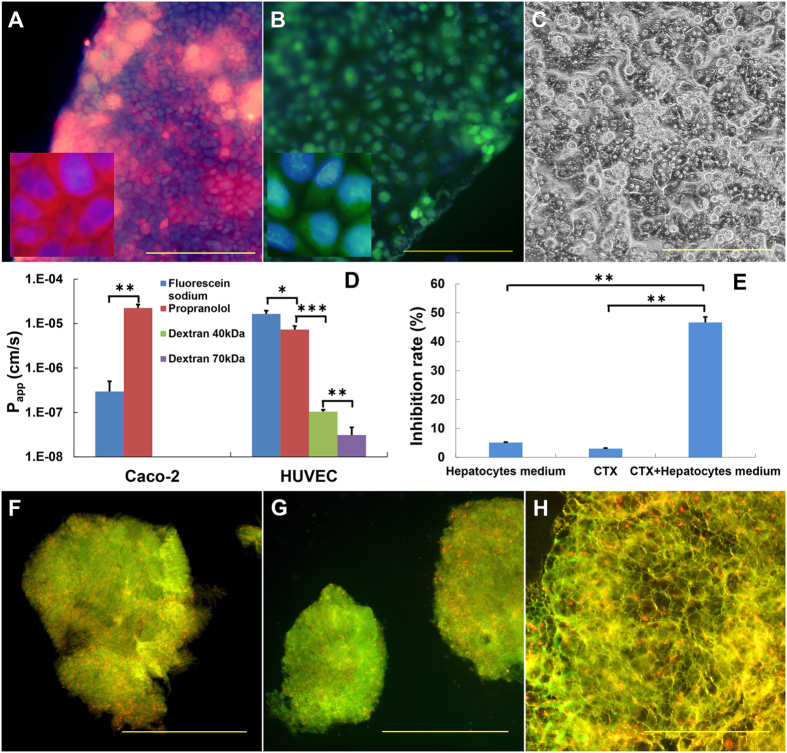
(**A**) The fluorescence image of the Caco-2 cell layer (stained with CelltrackerTM red and Hoechst 33342) on the porous membrane; (**B**) The fluorescence image of the HUVEC cell layer (stained with CelltrackerTM green and Hoechst 33342) on the porous membrane. (**C**) The bright field image of hepatocytes on the porous membrane. (**D**) The apparent permeability (P_app_) of soluble reagents with different molecular weights and properties through the Caco-2 cell or HUVEC layer. (**E**) The inhibition of MCF-7 cell proliferation by hepatocytes in culture medium, cyclophosphamide (CTX) and CTX metabolites in the microfluidic device. Viability analysis of the heart (**F**), lung (**G**) and adipose (**H**) tissue according to a LIVE/DEAD^®^ Viability/Cytotoxicity kit. Dead cells produced bright red fluorescence. The scale bars in A, B and C are 200 μm and 500 μm in F, G; H.N = 4. Error bars represent the standard error of the mean (SEM) of four independents experiments. Two-tailed significance was set at *p < 0.05, **p < 0.001 and ***p < 0.0005.

The Caco-2 cell layer was responsible for the selective absorption of drug candidates. Caco-2 cells were cultured on collagen-type-I-coated porous membrane for 17 days to form a cell layer with tight junctions. The image of the Caco-2 cell layer is shown in Fig. 2A, and the cell state can be observed. To observe the absorption of drug candidates by the Caco-2 cell layer, we applied propranolol and sodium fluorescein to the surface of the Caco-2 cell layer and evaluated the quantity of reagents that penetrated through the Caco-2 cell layer.

Figure 2D shows that the P_app_ of propranolol is much higher than that of sodium fluorescein. Propranolol is a typical intestine-absorbed drug, whereas sodium fluorescein cannot be absorbed. These data successfully demonstrate the selective absorption of propranolol over the sodium fluorescein by the Caco-2 cell layer, which implies three facts: (1) our Caco-2 cell layer was robust in terms of the absorption of drug candidates; (2) tight junctions successfully formed between Caco-2 cells and thereby avoided the diffusive transportation of sodium fluorescein through the Caco-2 cell layer; (3) the sealing of the microdevice was satisfactory. We also compared the P_app_ of propranolol and sodium fluorescein in the microdevice with those in a traditional transwell chamber. Good agreement was observed, which further indicates the reliability of our microdevice in terms of absorption of drug candidates.

To reproduce endothelial barrier function, a HUVEC layer was generated in the microdevice under both the Caco-2 cell layer and the hepatocyte layer. We cultured the HUVECs on the porous PC membrane for 3 days to form tight junctions between HUVECs. The image of the HUVEC layer is shown in Fig. 2B. We then tested the barrier capability of the HUVEC layer by measuring the apparent permeability of propranol, sodium fluorescein, 40 kDa dextran and 70 kDa dextran. Figure 2D shows that the P_app_ of large molecules (dextrans) is much lower than that of the small molecules (propranolol and sodium fluorescein), and the P_app_ appeared to inversely correlate with the molecular weight. These data show that the HUVEC layer was selectively permeable according to the molecular weight. This behavior is similar to that of capillaries *in vivo*. Figure 2D shows two additional facts: (1) tight junctions successfully formed between HUVECs and avoided the diffusive mass transfer through the HUVEC layer; (2) the sealing of the microdevice was satisfactory.

Hepatocytes in the microdevice are responsible for the metabolism of drug candidates. Unlike intestinal epithelial cells or vascular endothelial cells, the sinusoids are distributed through hepatic lobules to facilitate efficient mass transfer *in vivo*. Therefore, we were able to control the density of hepatocytes with this microdevice and ensure that the hepatocytes were sparsely distributed on the membrane (Fig. 2C). We used primary hepatocytes instead of cell lines to enhance the metabolism on the chip and prepared two layers of hepatocytes in the microdevice. Furthermore, to test the metabolism of primary hepatocytes, we tested the antitumor activity of cyclophosphamide (CTX) on primary hepatocytes cultured in a 35 mm-dish using CTX and cell culture medium as controls. CTX is an innocuous pro-drug that is transformed into aldehyde phosphnboramide in the liver and subsequently degraded into active phosphoramide mustard in tumor cells to inhibit their proliferation. Figure 2E shows significant variation between the CTX incubated with hepatocytes and the controls, proving the metabolic capability of our primary hepatocytes. Therefore, our primary hepatocytes can metabolize drug candidates: only the CTX incubated with hepatocytes showed antitumor activity, whereas CTX that had not been metabolized by the hepatocytes did not demonstrate this activity.

Additionally, we deposited heart, lung and fat tissues on a dialysis membrane assembled with the 5^th^ PDMS spacer to evaluate the distribution of drug candidates. Experimentally, we sealed these tissues in the microdevice for 72 h in a cell incubator and then stained them with the LIVE/DEAD^®^ Viability/Cytotoxicity Kit. Figure 2F–H shows the images of the stained tissues. Red spots, indicating dead cells, were sparsely distributed in the tissues, and approximately <10% cells were estimated to be dead after 72 h of culture. These dead cells may interfere with the toxicity test for these tissues. However, in our design, these tissues were used only to test the distribution of the drug candidates. Therefore, we deemed a partial loss of cell viability in the whole tissue fragments to be acceptable.

### Artificial ADME Process

**Figure 3:**
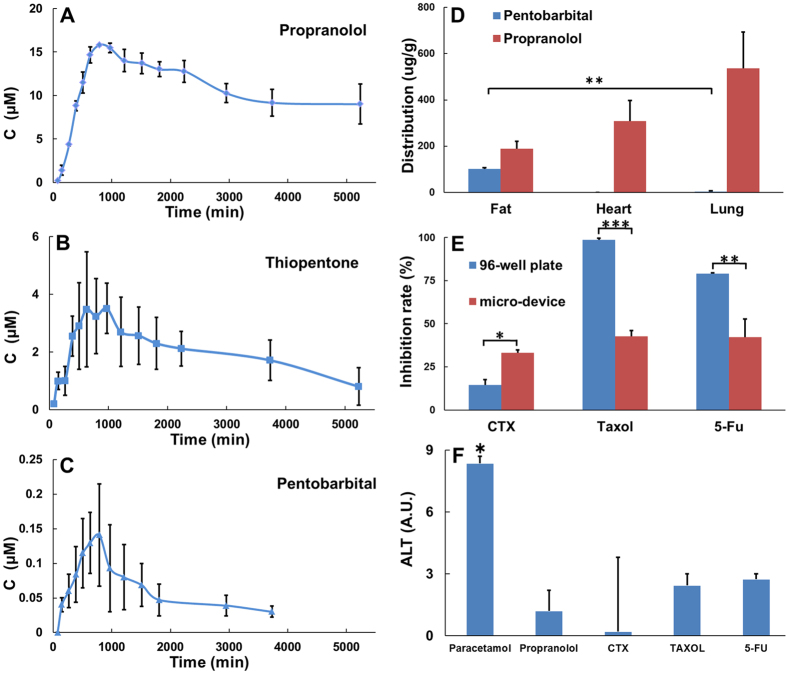
(**A–C**) Drug-time curves of propranolol, thiopentone and pentobarbital in the medium cycle in the fourth spacer. (**D**) The distribution of pentobarbital and propranolol in the heart, lung and adipose tissues. Pentobarbital was not detected in heart tissues in any of the three independent experiments. (**E**) The inhibition of MCF-7 cell proliferation in response to cyclophosphamide (CTX), taxol and 5-fuorouracil on the chip and in 96-well plate during 72 h of culture. (**F**) The alanine aminotransferase (ALT) values of paracetamol, propranolol, cyclophosphamide, taxol and 5-fuorouracil. N = 3. Error bars represent the standard error of the mean (SEM) of three independents experiments. Two-tailed significance was set to *p < 0.05 and **p < 0.001.

We applied the cell and tissue layers to the entire microdevice with spacers, as demonstrated in Fig. 1, and loaded the microdevice with drug candidates via the microchannel in the 1st spacer. The drug candidate solution covered the Caco-2 cell layer, which then absorbed the drug. The selective absorption of sodium fluorescein and propranolol by the Caco-2 cell layer is shown in Fig. 2D. The adsorbed drug candidate then passed through the HUVEC layer and diffused toward the hepatocyte layer, where it was then metabolized. The drug candidate and its metabolites then permeated through the HUVEC layer, diffused toward the MCF-7 cell layer, and then interacted with MCF-7 cell layer and the heart, lung and fat tissues. Finally, they were eliminated though the dialysis membrane. The entire artificial ADME process was characterized by the pentobarbital, thiopentone and propranolol concentration in the cell culture medium over time in the medium cycle in the 4th spacer (Fig. 3A–C). The concentration of the drugs first increased during the absorption process and then declined during the metabolism, distribution and elimination processes. This trend corroborated with *in vivo* observations^20^,^21^,^22^.

### On-chip pharmacokinetics

During the ADME process in the microdevice, we can measure a number of pharmacokinetic parameters on the chip, which is impossible in other reported *in vitro* drug screening platforms. First, the cell culture medium in the 4th spacer was sampled from its tank, and the concentration of drug candidates was plotted versus time. Figure 3A–C shows the drug-time curves of propranolol, thiopentone and pentobarbital in the microdevice. The graph suggests that the drugs were completely absorbed and eliminated. Additionally, quantitative information can be extracted from the curves. For example, the maximum concentrations of propranolol, thiopentone and pentobarbital were ~15.1, 3.8, and 1.14 μM, respectively. These data indicated that propranolol was better absorbed than thiopentone and pentobarbital. Although the molecular structures of thiopentone and pentobarbital differ by only one S atom, their drug-time curves significantly differed. Compared with pentobarbital, the additional sulfur atom renders thiopentone more lipid soluble, which resulted in better absorption. The qualitative and quantitative information from the drug-time curve in the microdevice can serve as a reliable reference for the design of animal tests^20^,^21^,^22^. An anomaly was also observed: the half-lives of pentobarbital, thiopentone and propanol appeared to be nearly identical and greater than 10 hrs. These half-lives should differ, and their similarity may result from the small mass transfer area of the dialysis membrane.

The heart, lung and fat tissues in the microdevice were able to interact with the drug candidates, and the distribution of drug candidates in these three tissues can be evaluated. Here, we demonstrate this function of the microdevice with pentobarbital and propranolol. We retrieved the heart, lung and fat tissues after testing and analyzed the remaining pentobarbital and propranolol in the tissues. Figure 3D shows that the propranolol was distributed across all the three tissues, and the its concentration decreased in the following order: lung > heart > fat. However, pentobarbital was detected in only the fat tissue. This result corroborates the results of animal testing^23^. Propranolol is preferentially deposited in the lung and heart tissues because it is a β-receptor blocker and tends to bind the β1-adrenergic receptor of the myocardium in the heart and the β2-adrenergic receptor of the bronchial smooth muscle in the lung.

### Estimation of the anti-tumor activity and hepatotoxicity

Because the tumor cells (MCF-7 cells) were integrated in the microdevice, we were able to estimate the anti-tumor activity of a drug candidate at a given pre-fixed concentration. Several classical anti-tumor drugs (CTX, taxol and 5-Fu) were used to demonstrate the feasibility of our working protocol in the microdevice. We set the concentration of CTX, taxol, and 5-Fu to 125, 5 and 55 μM, respectively, in accordance with the normalized doses used for clinical oral administration and injection. We then compared the anti-tumor activity of these drugs in the microdevice with those in a 96-well plate. Figure 3E shows that all the drugs demonstrated anti-tumor activity, but the anti-tumor activity in the 96-well plate and the microfluidic device significantly differed. CTX-induced tumor cell inhibition in the microdevice is ~6 times higher than that in the 96-well plate and this difference arises because the hepatocytes in the microdevice metabolize CTX, and these metabolites more effectively inhibit tumor cells than native CTX. Moreover, the tumor cell inhibition rates due to taxol and 5-Fu in the microdevice was ~2 times lower than those observed in the 96-well plate. This difference may be due to the significantly lower effective concentrations of taxol and 5-fu on the chip than in the 96-well plate due to the ADME process in the microdevice. In summary, the testing of anti-tumor activity in the microdevice more closely approximates the clinical situation than the 96-well plate due to the ADME process in the microdevice^24^. The testing results obtained from the microdevice may serve as a reliable reference for the design of animal tests.

The microdevice, which contained primary hepatocytes, allows the hepatotoxicity of a drug candidate to be assessed at a pre-defined concentration. In our study, paracetamol, propranolol, cyclophosphamide, taxol or 5-Fu was added to the microchannel on the 1^st^ spacer. After 48 h, the glutamic-pyruvic transaminase activity of cell culture medium incubated with hepatocytes in the 3^rd^ PDMS spacer was collected and detected using ALT kits. Figure 3F shows that all drugs exhibited hepatotoxicity, and the toxicity of paracetamol was extraordinarily high. This result agrees with those of animal tests^25^.

## Discussion

In this study, we stacked functional cells and tissues layer by layer in a microdevice. This modularized strategy is flexible and advantageous: (1) the quantity of cells or tissue deposited can be controlled by the membrane area; (2) the liquid volume between the cell layers can be controlled by the thickness of the spacer; (3) the number of cell types that can be integrated in a single chip is virtually unlimited because they are relatively independent units vertically separated by porous membranes and PDMS spacers; (4) functional cells and tissues, as well as the cell culture medium, can be easily retrieved by simply disassembling the device; therefore, the bioactivity of each cell type and the communication between any two connected cell layers can be readily characterized by an off-line analysis of retrieved samples, such as high-resolution optical imaging; (5) the combinations of functional cells are highly flexible. For example, we can simply combine Caco-2 cells and tumor cells to investigate the effect of drug absorption on antitumor efficacy. We can also combine Caco-2 cells and hepatocytes to investigate the effect of drug absorption on toxicity. We fabricated an endothelium barrier other than an endothelium-lined vasculature in the device because it simplified device fabrication and operation.

The highlight of our microdevice is its ability to recreate the drug ADME process. The ability of a compound to ultimately be developed into a successful drug depends largely on the ADME process of this drug *in vivo*. The ADME process is traditionally evaluated using laboratory animals, which is an expensive and onerous process. Using our laminated chip to evaluate ADME properties of a candidate drug before an animal test would significantly reduce the number of animal tests thereafter, thus saving time and money.

Because it can recreate the drug’s ADME process, this microdevice performs in a manner similar to a laboratory SD rat in preclinical tests and demonstrates unparalleled advantages over traditional *in vitro* drug screening tools. First, the drug-time curve can be obtained from the laminated chip (Fig. 3A–C), which is impossible with traditional *in vitro* drug screening tools. Second, the drug distribution can be evaluated based on the microdevice (Fig. 3D), which is also impossible with traditional *in vitro* drug screening tools. Third, the efficacy test results obtained using the microdevice, which are similar to those from animal and clinical studies, are highly influenced by the ADME processes and differ significantly from the results obtained using a 96-well plate (Fig. 3E). Fourth, the administration method of a drug can be predicted using the laminated chip, but not with *in vitro* drug screening tools. To demonstrate the antitumor activity of taxol, its IC_50_ concentration in the microdevice (up to 5 μM) was determined and was much higher than that in a 96-well plate (<2 nM) due to the poor intestinal permeability, indicating that taxol can be administered only by injection and not orally. In fact, taxol is clinically administered only by injection^22^. Fifth, because our device is similar to a laboratory SD rat, it can provide abundant information for drug development. In addition to the drug-time curve, distribution, and efficacy testing mentioned above, our device can also be used to conveniently evaluate the absorption, metabolism and toxicity.

Notably, we aimed to recreate the ADME process *in vitro*, not to recreate the living body of an animal. Thus, our device does not mimic the living body. For example, it does not contain a complex vascular network to connect and feed different types of functional cells. The device also does not lend itself to allometric scaling, and the size ratio between different functional cells does not follow the conditions *in vivo*; the area of the Caco-2 cell layer and dialysis membrane is relatively small, resulting in a small mass transfer rate, among other effects. However, our device transported drugs out through the cell barriers and tissues in the following order: intestinal wall - vessel barrier - liver - vessel barrier - different organs – kidney (dialysis membrane). This device allowed the investigation of the mass transfer property of drugs *in vitro*.

Technically, the co-culture of several dense cell layers and living tissue figments in a narrow space consumes large amounts of nutrients. Three culture medium cycles containing different components were supplied into the 1st, 4th and 6th PDMS spacers at a flow rate of 5 μl/min, generating a max fluidic shear of less than 10 dyne at channel restriction. The flow rate was determined based on two factors. First, when the three culture media flow at different rates, the resultant pressure difference may give rise to ultrafiltration that causes errors in the experiment. Second, the MCF7 cells cultured on the PC member coated with collagen suffer from fluidic shear and may detach at higher flow rates.

In conclusion, a laminated microdevice integrating multiple cells and tissues was developed. Using our protocol, various cell types can be integrated in a single laminated chip, and the arrangement of functional cells and tissues in the microdevice is also highly flexible. This device is expected to serve as an alternative to the laboratory SD rat for the comprehensive evaluation of drug candidates. Furthermore, all animal cells and tissues used in the demonstrated device can likely be replaced by cells and tissues of human origin to further increase the value of this device in various fields, such as drug discovery, personalized medicine, toxin testing, etc.

## Methods

### Device assembly and operation

Prior to assembly, the PDMS plates were sterilized with 75% alcohol and dried on a clean bench under ultraviolet irradiation. From the top, functional cells, including Caco-2 cells, human umbilical vein endothelial cells (HUVEC), primary hepatocytes and Michigan Cancer Foundation-7 (MCF-7) cells, were cultured on an individual porous polycarbonate membrane (Whatman, USA) with a 1-μm pore size, respectively. The heart, lung, and fat tissues were individually deposited on the dialysis membrane (MWCO:50000, Spectrum, USA). The membrane-based cell and tissue layers were assembled along the vertical direction with the aid of PDMS plates, as shown in Fig. 1. The spaces inside the 2^nd^, 3^rd^ and 5^th^ PDMS spacer were filled with cell culture medium during assembly. Ultimately, the PMMA frame was covered, and all screws were tightened to seal the entire device.

The tubing was connected to the inlet and outlet holes at the top of the PMMA frame. The culture medium was infused into the inlets from one of three medium tanks with a multi-channel peristaltic pump (205 S/CA12, WATSON MARLOW, British). Each culture medium contained different components and was supplied into the 1^st^, 4^th^ or 6^th^ PDMS spacer at a flow rate of 5 μl/min. In detail, the cell-culture medium consisting of DMEM/High Glucose (1x) medium (Hyclone, USA), 10% fetal bovine serum (FBS) (GIBCO, USA) and 1% non-essential amino acids (NEAA) (GIBCO, USA) was fed into the microchannel on the 1^st^ spacer; the culture medium containing DMEM/high glucose(1x) supplemented with 10% fetal bovine serum, 10 ng/ml EGF, 10 mM nicotinamide and 1% ITS-X (GIBCO, USA) was fed into the microchannel on the 4th spacer; DMEM/F12 medium (Hyclone, USA) was fed into the microchannel on the 6^th^ spacer. Additionally, the drugs and reagents used in this study were completely dissolved in tank of the 1^st^ culture medium and fed into the device by a peristaltic pump for the pharmacokinetic and toxicity studies. The overall operation time was 72 hours, during which all cells and tissues in the microdevice remained viable.

Non-uniform tubing was used to avoid the generation of air bubbles in the device (the set-up is described in detail in the S.I.). After operation, the fluids were recycled into the culture medium tanks via the outlets at the top of the PMMA plate.

### Isolation of primary hepatocytes and tissues

Primary hepatocytes were isolated from male SD rats (weighing 180–220 g) according to a modified two-step method described by Seglen^26^. The viability of hepatocytes was >85% based on Trypan blue exclusion. The heart, lung and fat tissues were also excised from the male SD rats. Fresh tissues less than 1 mm^3^ in volume were minced into fragments on ice, resuspended in cold PBS buffer, and centrifuged 3 times to remove the blood. The tissue fragments were filled in the central holes (2-mm diameter) of the 5^th^ PDMS spacer separately before assembly, and weighed respectively before the detection of propranolol and pentobarbital in the tissue. The primary hepatocytes and tissues were isolated in accordance with the laws set forth by the Chinese government and the regulations of the State Project for Essential Drug Research and Development in China. All experimental protocols were approved by the Specific Pathogen Free Animal Laboratory of the Dalian Medical University in China.

### Cell culture

The Caco-2 intestinal epithelial cells were cultured in DMEM/High Glucose medium (1x) containing 10% fetal bovine serum (FBS) and 1% non-essential amino acids (NEAA). HUVEC and MCF-7 cells were cultured in DMEM/F12 medium containing 10% fetal bovine serum. The primary hepatocytes were cultured in DMEM/high glucose (1x) medium supplemented with 1% fetal bovine serum, 10 ng/ml EGF, 10 mM nicotinamide and 1% ITS-X. 100 units/ml of penicillin and 100 mg/ml of streptomycin (Hyclone, USA) were added to all the aforementioned media.

Porous polycarbonate membrane was cut into round pieces with a diameter of 7 mm, sterilized with 75% alcohol. Dried pieces were arranged in 35 mm-dishes and coated with different concentration of type-I collagen (Lifetech, USA.), 5 μg/cm^2^ for HUVEC and Caco-2 cells, and 40 μg/cm^2^ for hepatocytes and MCF-7 cells. The Caco-2, HUVEC , MCF-7 cells (harvested with trypsin (0.25%)/EDTA (0.02%) solution) and fresh primary hepatocytes were cultured in 35 mm-dishes at concentrations of 8 × 10^4^/cm^2^, 1 × 10^5^/cm^2^ and 3 × 10^4^/cm^2^ and 8 × 10^4^/cm^2^, respectively.

The 35 mm-dishes containing porous membranes with cells were maintained at 37 °C in a humidified incubator under 5% CO_2_ in air. The porous membranes bearing hepatocytes and MCF-7 cells were assayed after 1 day of culture. HUVEC and Caco-2 cells were seeded on porous membranes for 3 days and 17 to 20 days, respectively, to ensure maturity and tight junctions. Then, the porous membranes loaded with cells were taken out and assembled with PDMS spacers.

### Characterization of cell layers and tissue

The absorption and barrier capacities of the Caco-2 cell and HUVEC layers were evaluated by measuring the apparent permeability (P_app_) of propranolol (Sigma, USA), sodium fluorescein (Sigma, USA), and labeled dextran (FITC-dextran, MW 40 kDa and Rhodamine B-dextran, MW 70 kDa, Sigma, USA) through cell layer. The cell layer was clamped by two PDMS spacers and fastened with PMMA plates. One milliliter of D’Hanks solution containing propranolol (240 μM), sodium fluorescein (2 mg/ml), FITC-dextran (1 μM) or Rhodamine B-dextran (1 μM) was perfused through the microchannel of the upper PDMS layers, and blank D’Hanks solution was circulated through the lower microchannel at a flow rate of 5 μl/min. We then sampled and detected the propranolol and sodium fluorescein in the lower circulating solution every 30 mins. and P_app_ was calculated using equation 1:


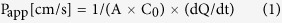


where A = area of mass transfer, C_0_ = donor concentration of reagent in upper medium, and dQ/dt = transmembrane transportation rate.

To measure the metabolism in the hepatocytes, 400 μl of hepatocyte culture medium containing 125 μM of CTX (Sigma, USA) was co-incubated with the hepatocytes in a 24-well plate at 37 °C for 12 h. One hundred microliters of medium was then sampled and added to a 96-well plate with containing MCF-7 cells and 100 μl of fresh MCF-7 cell culture medium. Moreover, 100 μl of blank hepatocyte culture medium or medium containing 125 μM CTX was added to a 96-well plate containing MCF-7 cells and 100 μl of fresh MCF-7 cell culture medium. After 48 h, the proliferation of MCF-7 cells in all three 96-well plates was evaluated with an MTT assay.

To evaluate viability of tissues, the chip was assembled and operated at 37 °C for 72 h. The tissue fragments were then separated and stained with a LIVE/DEAD^®^ Viability/Cytotoxicity kit according to the manufacturer’s instructions.

### Illustration of the drug-time curve

Propranolol, thiopentone (Wanjiashou bio-tech co., LTD, China) and pentobarbital (Wanjiashou bio-tech co., LTD, China) were added to the microfluidic device from the medium cycle into the first space at a concentration of 240 μM. A 20-μl sample was collected from the storage tank of the medium cycle in the fourth spacer at each time point, mixed with 20 μl of methanol and centrifuged at 12,000 rpm. The samples were then analyzed by HPLC (Agilent 1100 Series) with Agilent ZORBAX SB- C18 (250 mm × 4.6 mm, 5 μm) columns. For propranolol, the mobile phase was methanol-water-KH_2_PO_4_(0.2 mol/l) (55:43:2, v/v), and detection was carried out at 210 nm. For thiopental and pentobarbital, the mobile phase was methanol-water (1:1, v/v), and detection was carried out at 240 nm and 220 nm, respectively.

### Evaluation of distribution

The same concentration of propranolol and pentobarbital (240 μM) was added to the microfluidic device from the medium cycle to the first spacer for 48 h. The tissue fragments were retrieved from the microfluidic device, washed 3 times with PBS, and dried with filter paper. After weighing, the tissue fragments were resuspended in 200 μl of a water-methanol solution (1:1, v/v), lysed with ultrasound, and filtered. The propranolol and pentobarbital in the filtrate were analyzed by HPLC as described above.

### Evaluation of antitumor activity

To evaluate the antitumor activity, 125 μM cyclophosphamide, 5 μM taxol or 55 μM 5-fuorouracil was added to the microfluidic device via the medium cycle in the first spacer. As a control, MCF-7 cells in 96-well plates were exposed to the same concentration of the three types of drugs. After 72 h, the MCF-7 cell layers were retrieved from the chip, and the proliferation of MCF-7 cells in both chips and the 96-well plates were evaluated with an MTT assay. A device with blank medium were used as a control.

### Evaluation of hepatotoxicity

Paracetamol, propranolol, CTX, taxol or 5-fuorouracil was added to the microfluidic device from the medium cycle in the first spacer at the same concentration (240 μM) for 48 h, and the blank medium cycle was used as a control. The hepatocyte culture medium was retrieved from the chip, and the activity of glutamic-pyruvic transaminase was detected with an alanine aminotransferase (ALT) analysis kit (Biosino, Shanghai, China).

## Additional Information

**How to cite this article**: An, F. *et al*. A Laminated Microfluidic Device for Comprehensive Preclinical Testing in the Drug ADME Process. *Sci. Rep*. **6**, 25022; doi: 10.1038/srep25022 (2016).

## Supplementary Material

Supplementary Information
