# Supplementary material for: A Laminated Microfluidic Device for Comprehensive Preclinical Testing in the Drug ADME Process
Source: Sci Rep. 2016 Apr 28;6:25022. doi: 10.1038/srep25022 (PMC4848557; doi:10.1038/srep25022)
Supplement: Supplementary Information [file srep25022-s1.doc]

**Supporting Information of**

**“A Laminated Microfluidic Device for Comprehensive Preclinical Testing in the Drug ADME Process”**

**Fan An, Yueyang Qu, Yong Luo, Ning Fang, Yang Liu, Zhigang Gao, Weijie Zhao, Bingcheng Lin**


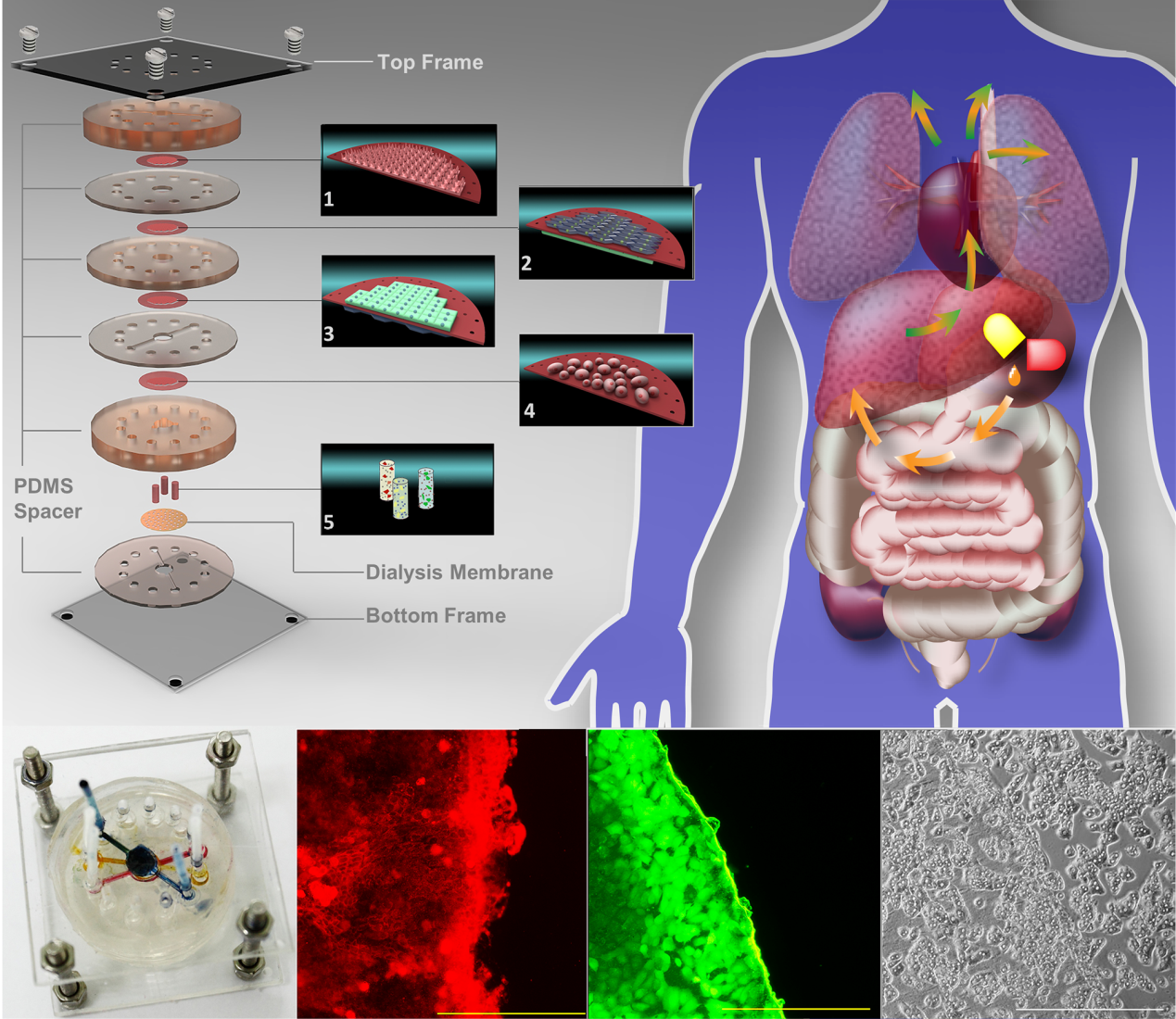


Figure S1 The photo image of our 3D chip device, in which the microhannels are highlighted by the blue, yellow and red dyes, respectively.


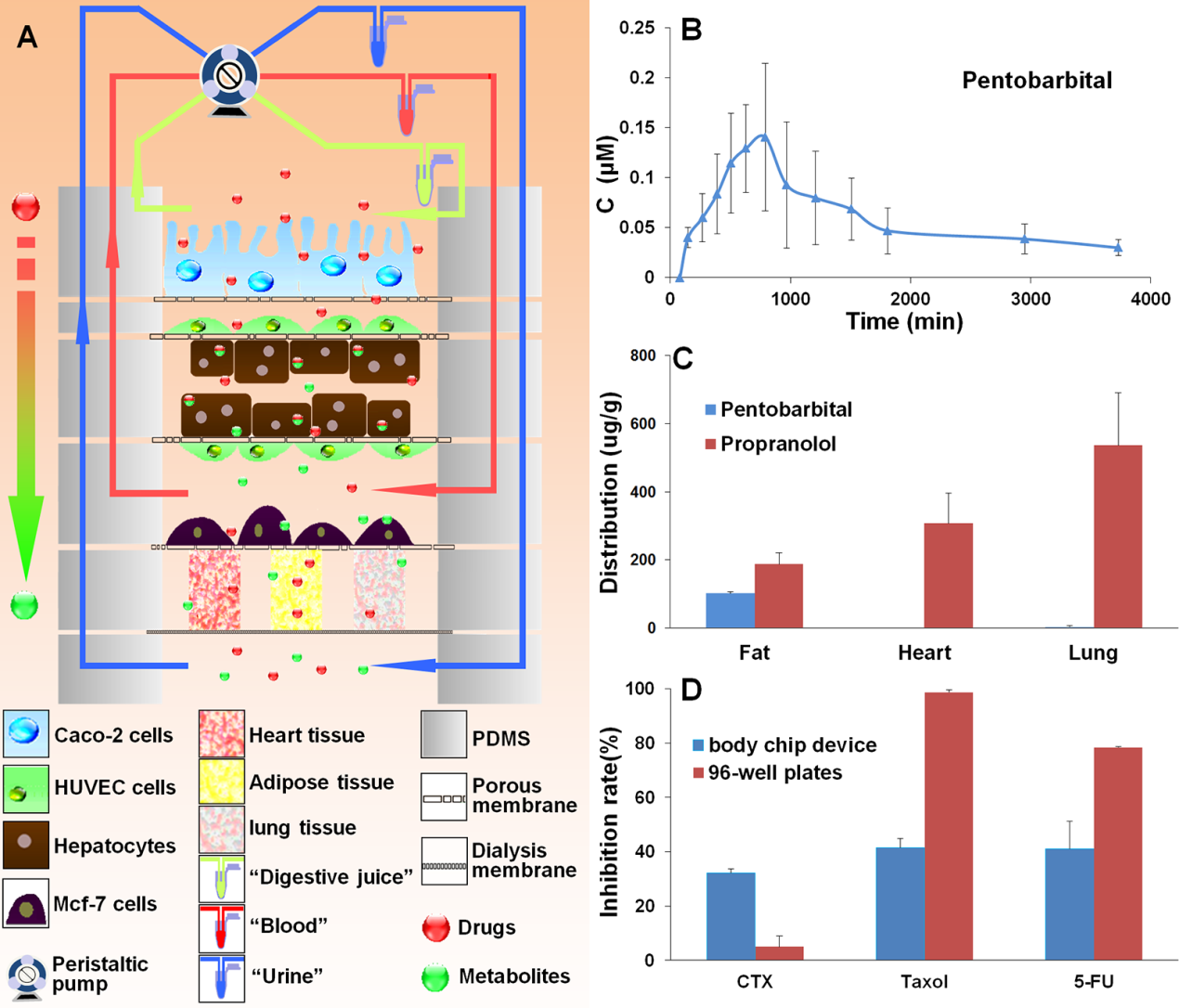


Figure S2 The longitudinal-section view of the microdevice. The arrow at the very left indicates the evolution of a drug in our organ chip device. The arrows in red, yellow, and blue indicates the direction of cell culture medium perfusion.


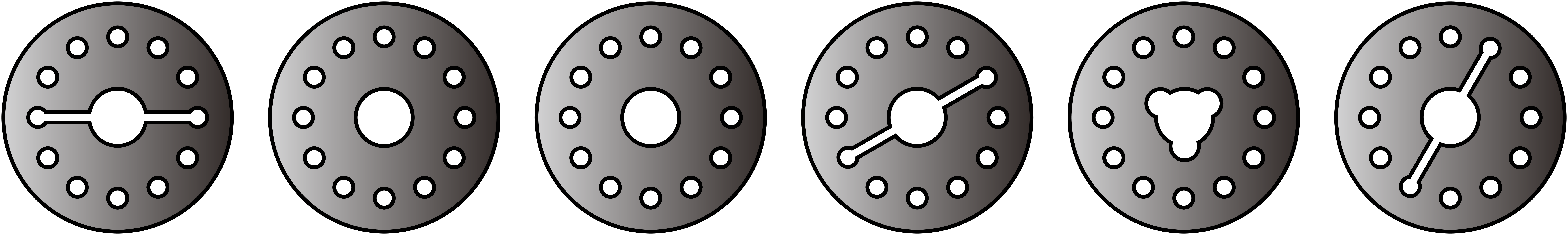


Figure S3 The vertical view of the six PDMS spacers, (from the left to right, 1^st^-6^th^)


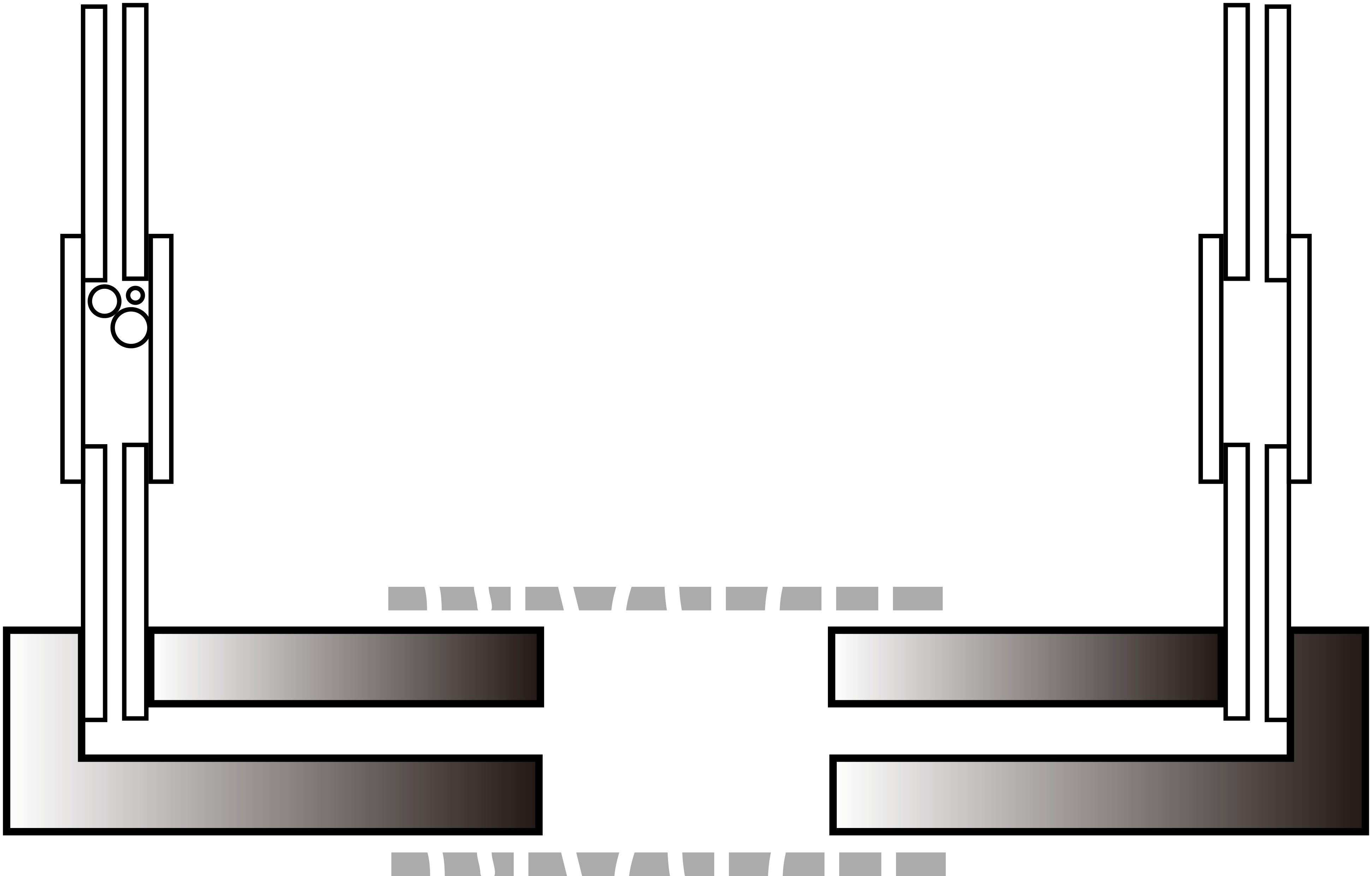


Figure S4 The detailed structure of PDMS spacer with tubings in. A connector with bigger inner diameter was designed to trap air bubbles. The dashed grey lines indicated the cell-loaded membrane.
